# Supplementary figures and images for: Multi-omics data integration reveals metabolome as the top predictor of the cervicovaginal microenvironment
Source: PLoS Comput Biol. 2022 Feb 23;18(2):e1009876. doi: 10.1371/journal.pcbi.1009876 (PMC8901057; doi:10.1371/journal.pcbi.1009876)

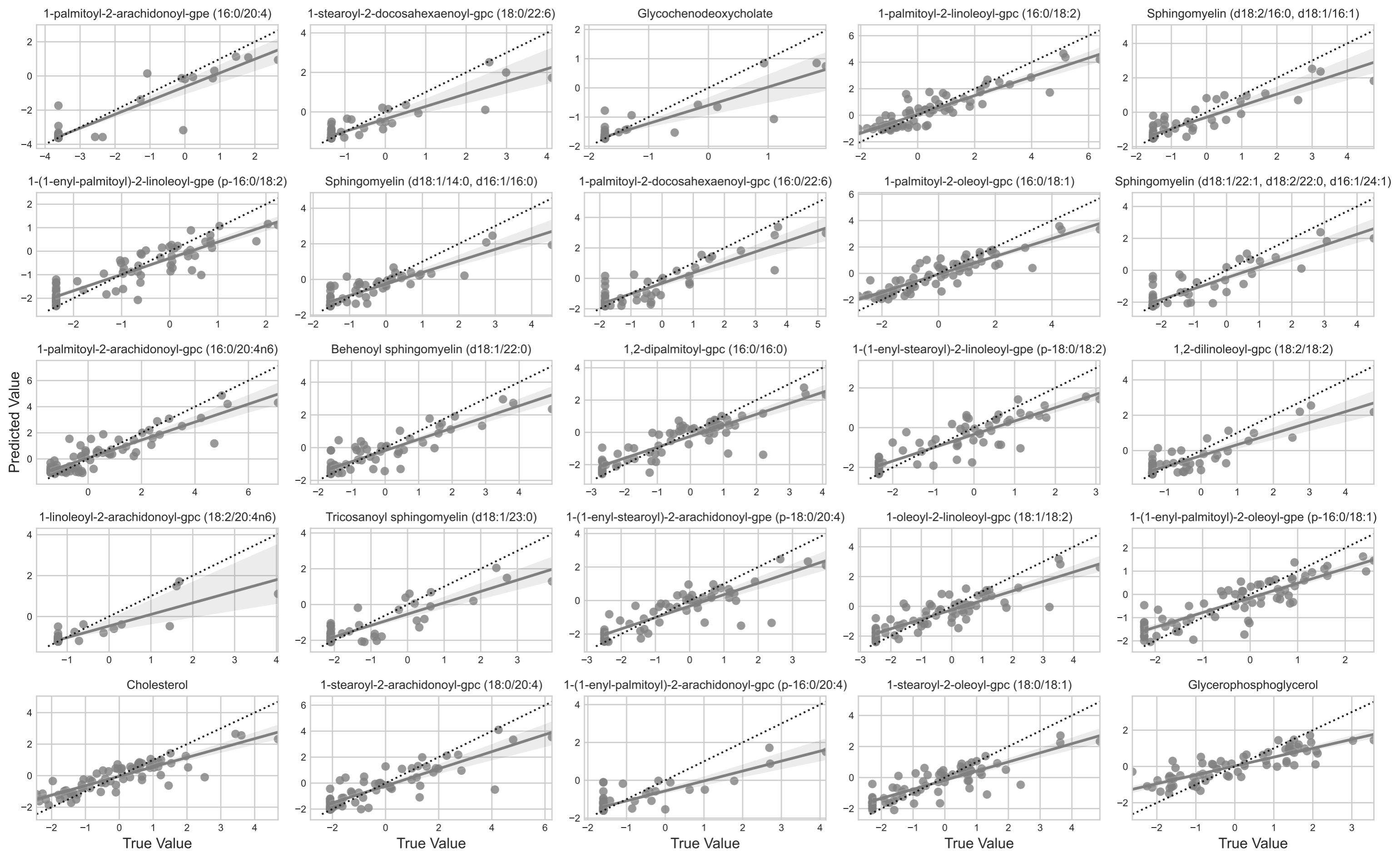

Supplement: S1 Fig — Random Forest regressors with 10-fold cross-validation were used to predict the abundance of each selected metabolite in S1 Table based on combined microbiome and immunoproteome datasets. Scatterplots display the linear regression of predicted vs. true log concentrations for the top 20 most accurately predicted metabolites. Dotted lines indicate an ideal 1:1 slope. Grey lines and shading indicate the regression trend line and 95% CI. (PDF) [file pcbi.1009876.s003.pdf]

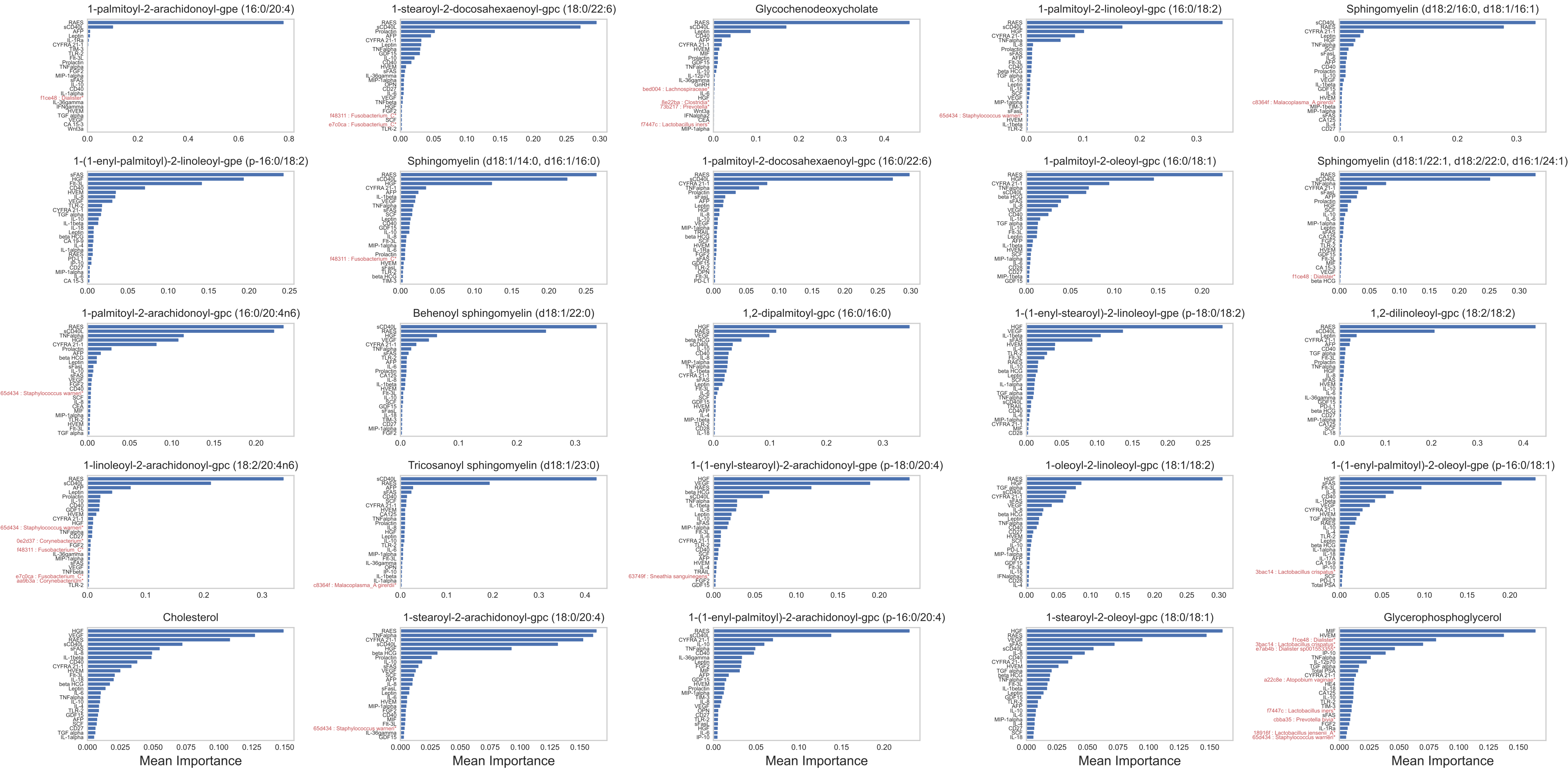

Supplement: S2 Fig — Feature importance of top 15 features used in the final Random Forest regression model for each metabolite prediction displayed in S1 Fig. *Microbial features are displayed in red, with the first 6 characters of the ASV ID followed by the genus/species-level Greengenes taxonomy. (PDF) [file pcbi.1009876.s004.pdf]

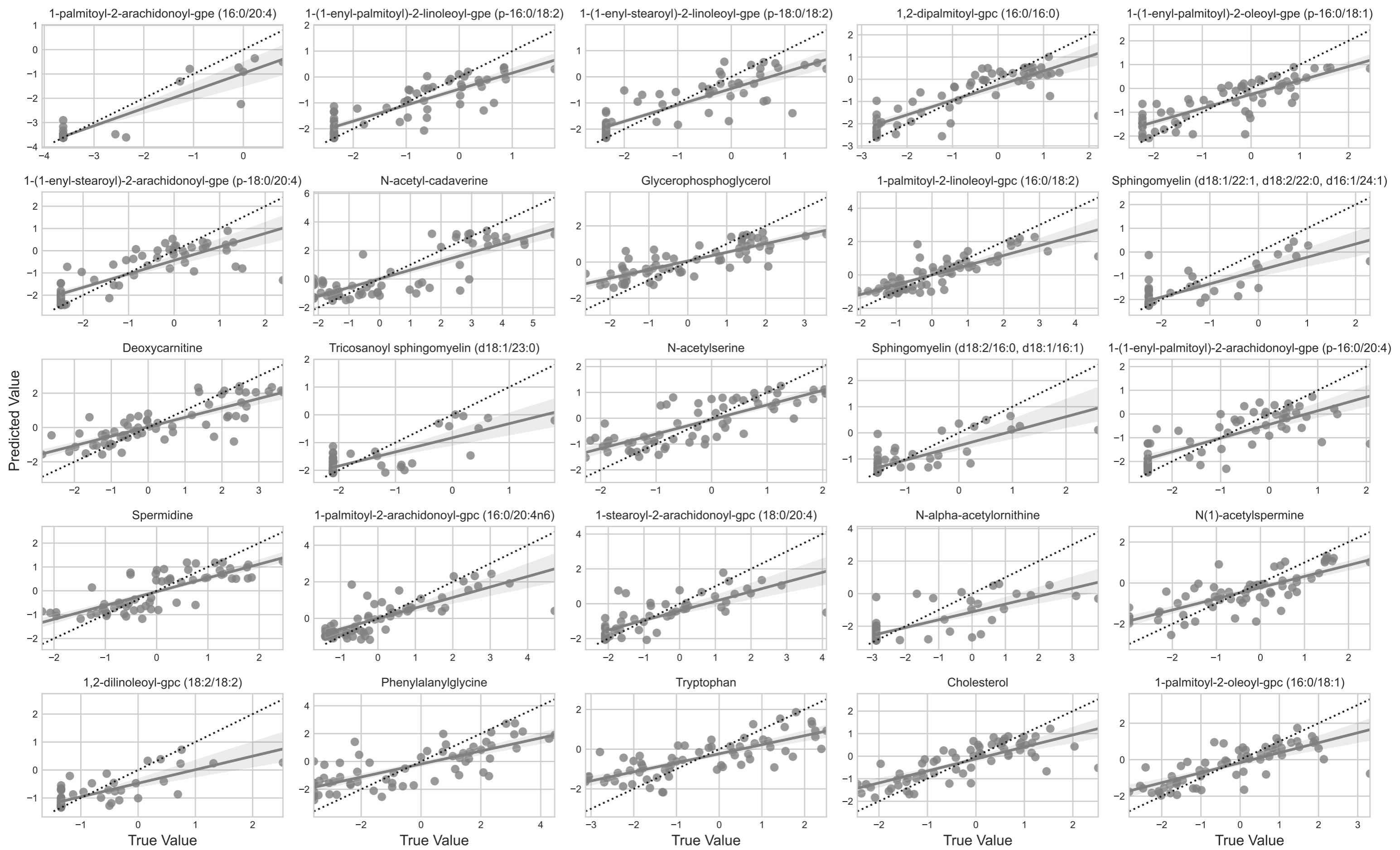

Supplement: S3 Fig — Plots display the predictive accuracy of the top 20 metabolites displayed in S1 Fig, but with cancer cases removed. Predictive accuracy remains high for most metabolites, indicating that cancer cases do not drive the associations observed for that metabolite. (PDF) [file pcbi.1009876.s005.pdf]

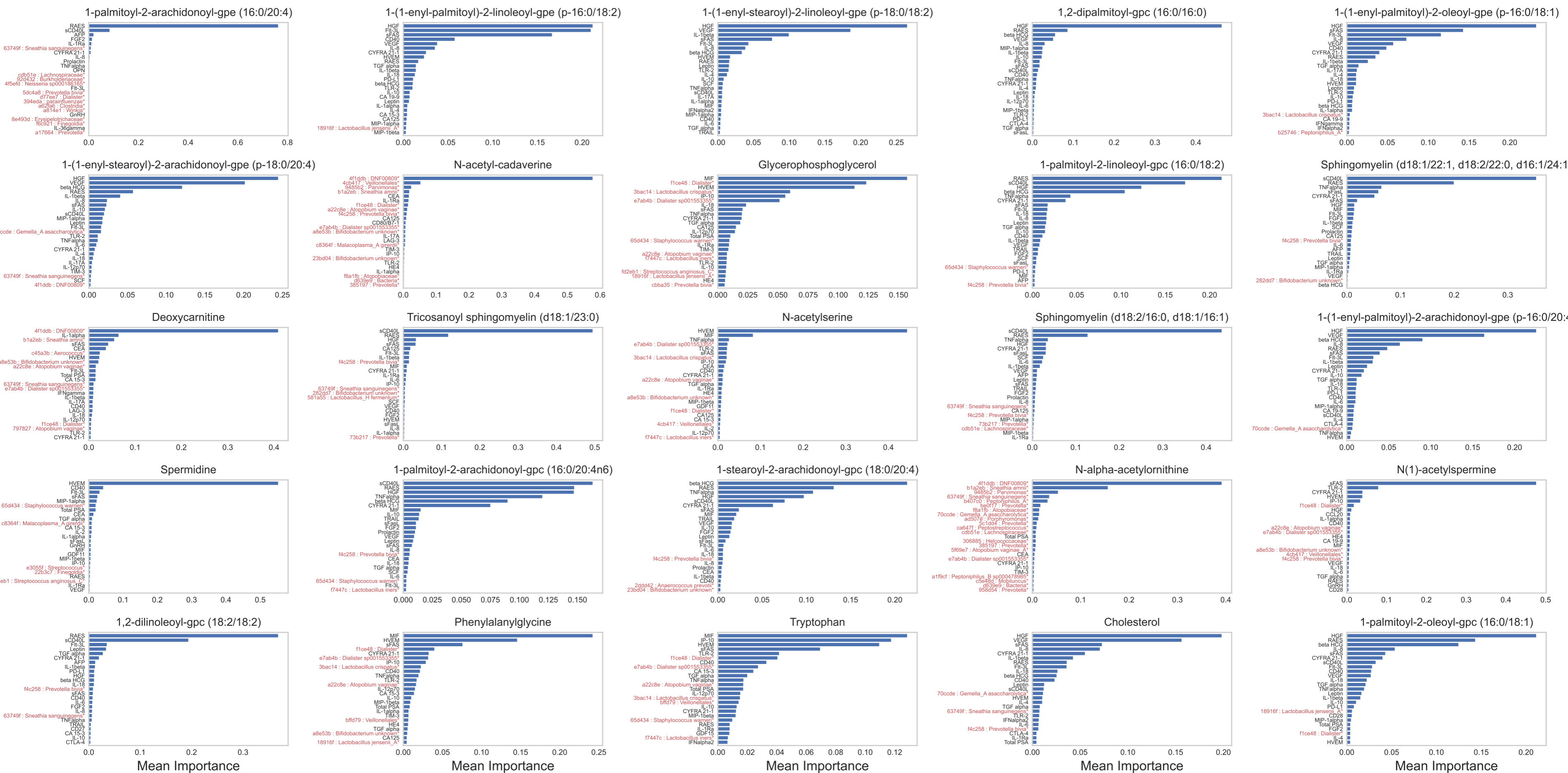

Supplement: S4 Fig — Feature importance of top 15 features used in the final Random Forest regression model for each metabolite prediction displayed in S3 Fig. *Microbial features are displayed in red, with the first 6 characters of the ASV ID followed by the genus/species-level Greengenes taxonomy. (PDF) [file pcbi.1009876.s006.pdf]

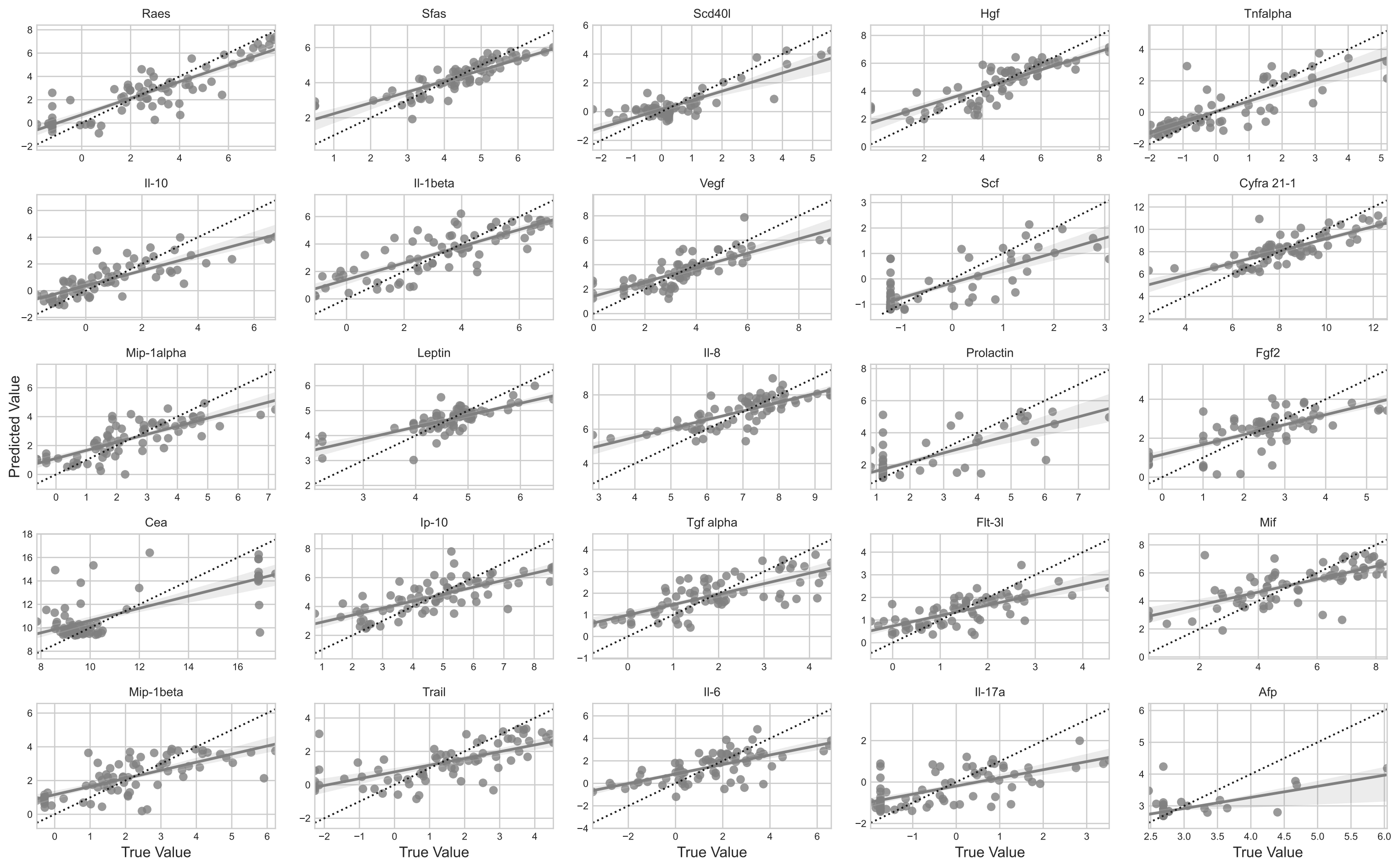

Supplement: S5 Fig — Random Forest regressors with 10-fold cross-validation were used to predict the abundance of each selected biomarker in S2 Table based on combined microbiome and metabolome datasets. Scatterplots display the linear regression of predicted vs. true log concentrations for the top 20 most accurately predicted biomarkers. Dotted lines indicate an ideal 1:1 slope. Grey lines and shading indicate the regression trend line and 95% CI. (PDF) [file pcbi.1009876.s007.pdf]

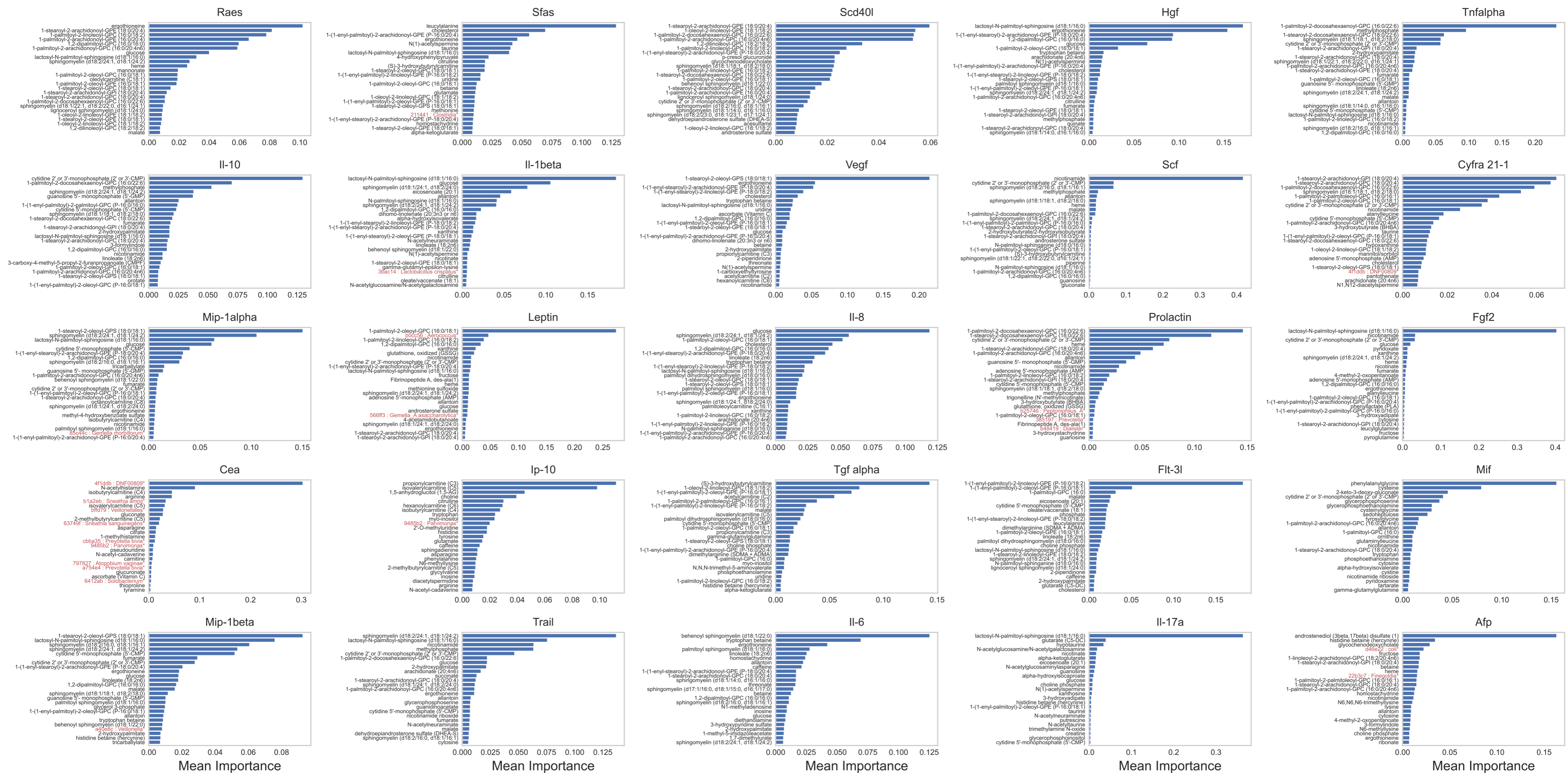

Supplement: S6 Fig — Feature importance of top 15 features used in the final Random Forest regression model for each cancer biomarker prediction displayed in S5 Fig. *Microbial features are displayed in red, with the first 6 characters of the ASV ID followed by the genus/species-level Greengenes taxonomy. (PDF) [file pcbi.1009876.s008.pdf]

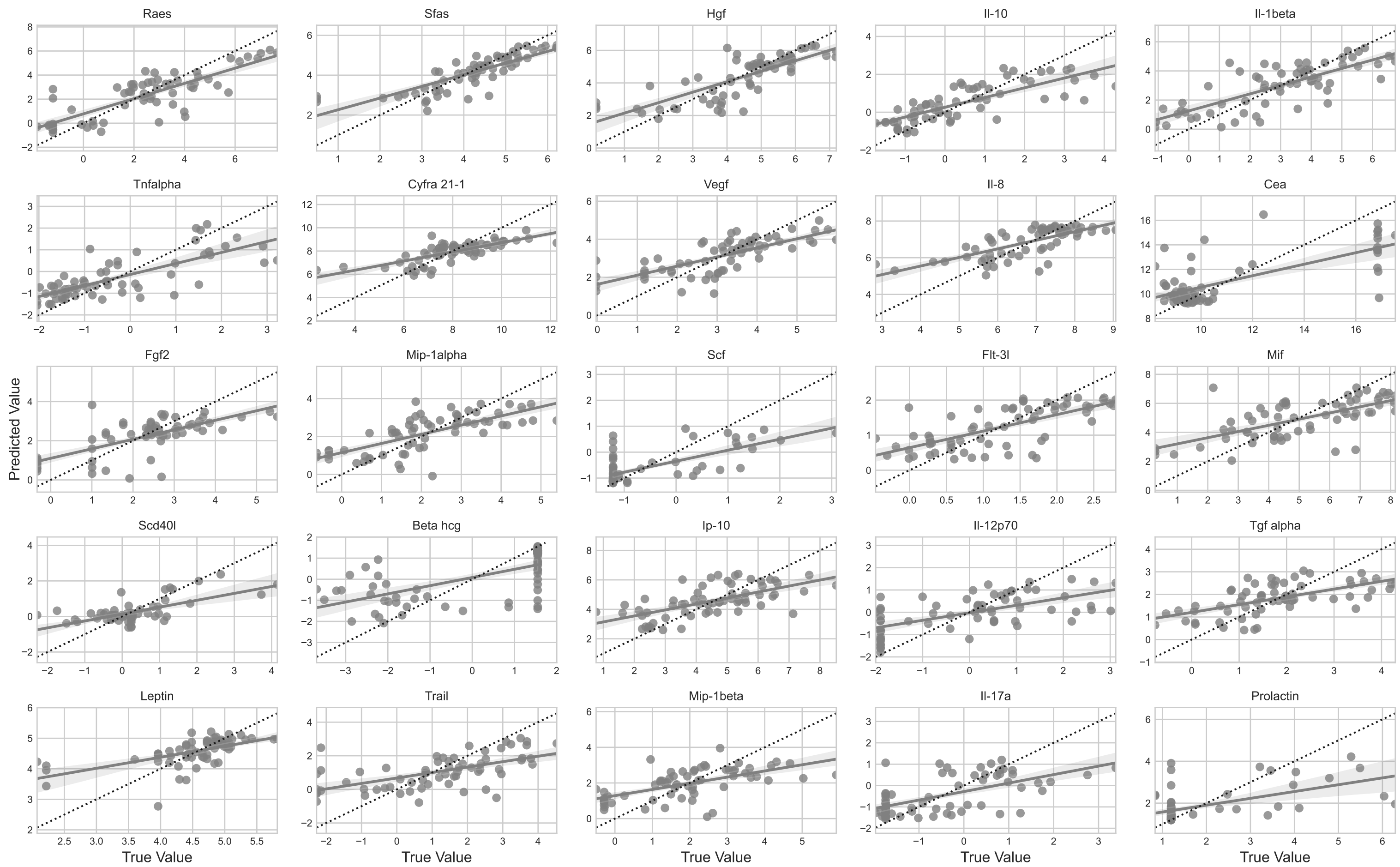

Supplement: S7 Fig — Plots display the predictive accuracy of the top 20 biomarkers displayed in S5 Fig, but with cancer cases removed. Predictive accuracy remains high for most metabolites, indicating that cancer cases do not drive the associations observed for that metabolite. (PDF) [file pcbi.1009876.s009.pdf]

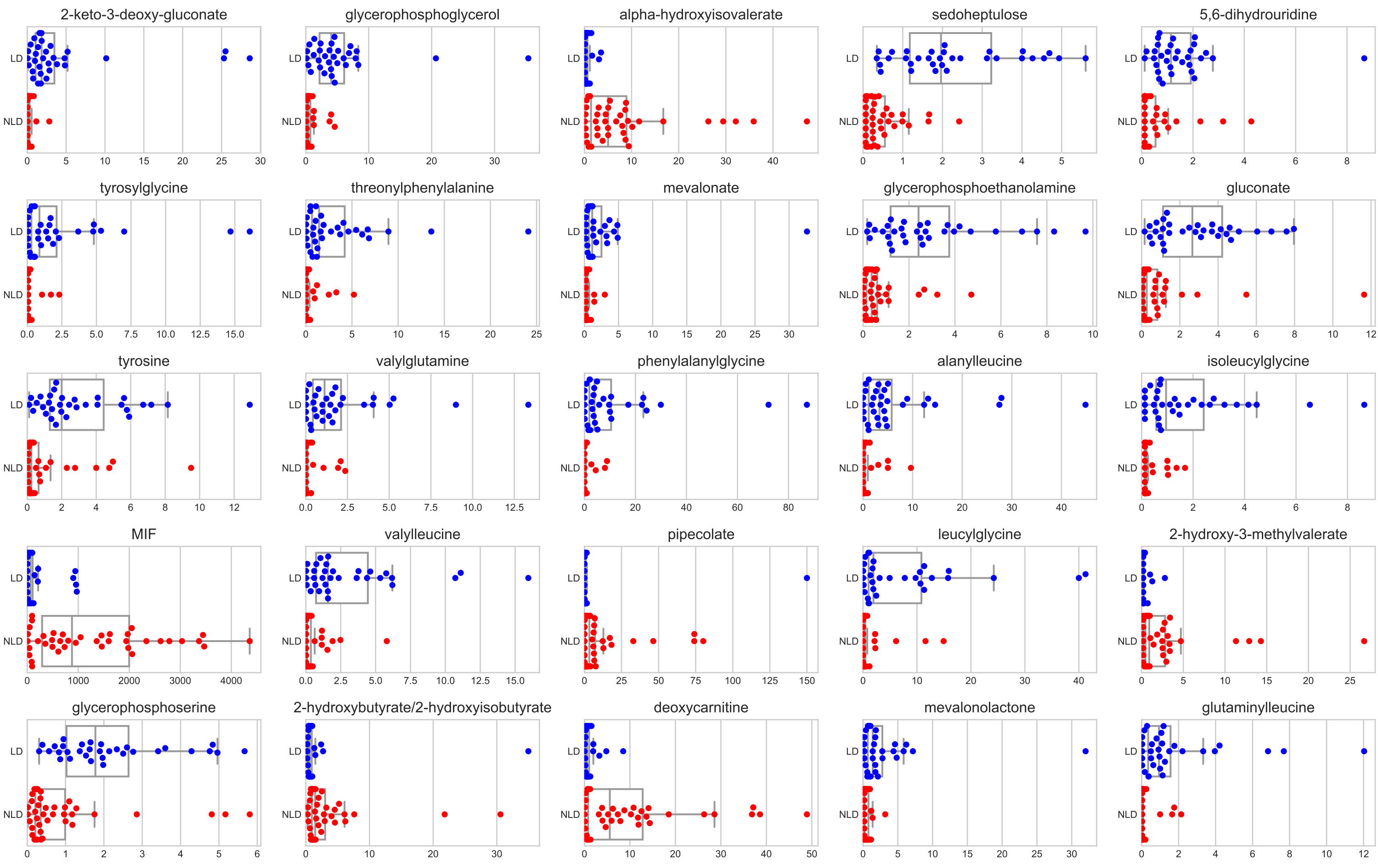

Supplement: S9 Fig — Boxplots display quartile distributions, swarmplots display individual values of top important feature abundances in LD and NLD groups. (PDF) [file pcbi.1009876.s011.pdf]

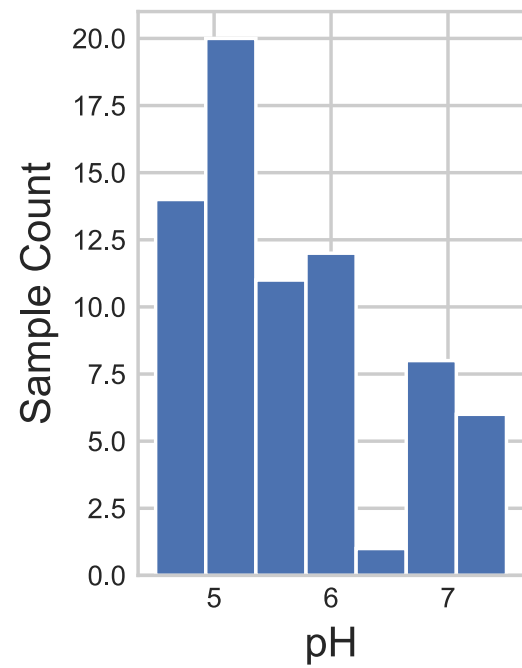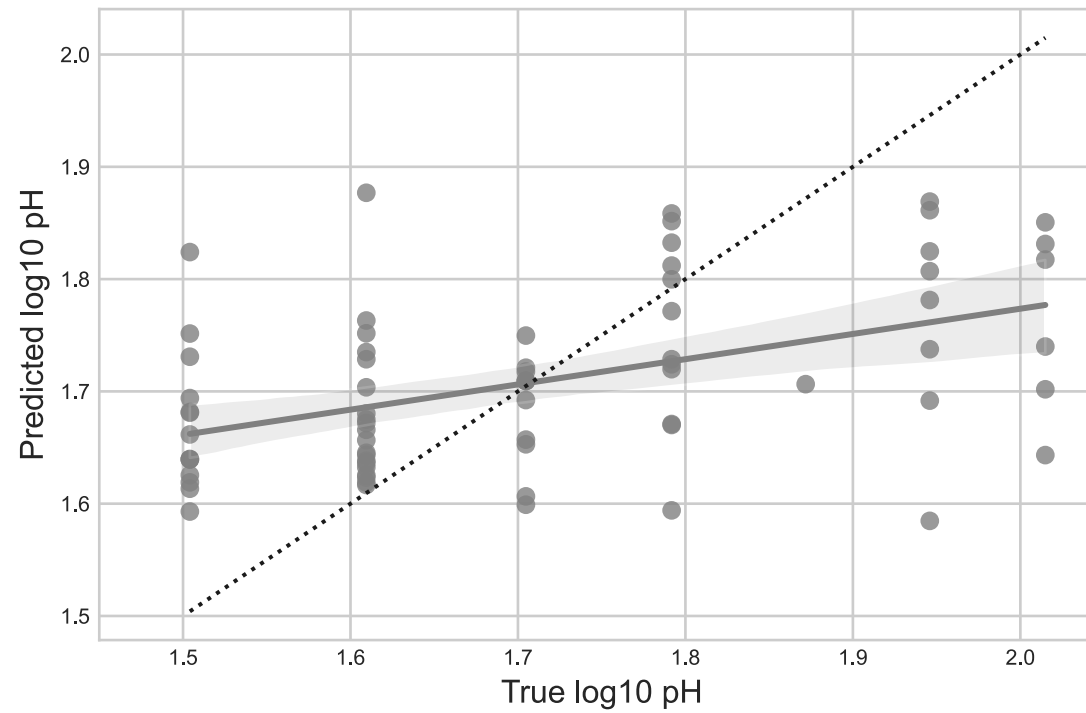

Supplement: S10 Fig — Left, histogram displays number of samples per pH value, binned into increments of 0.5 (min = 4.5, max = 7.5). Right, scatterplot displays true vs. predicted log10 vaginal pH for each subject (using 10-fold cross-validation random forest regressors to predict vaginal pH across subjects), indicating very poor regression results due to pH skew. (PDF) [file pcbi.1009876.s012.pdf]

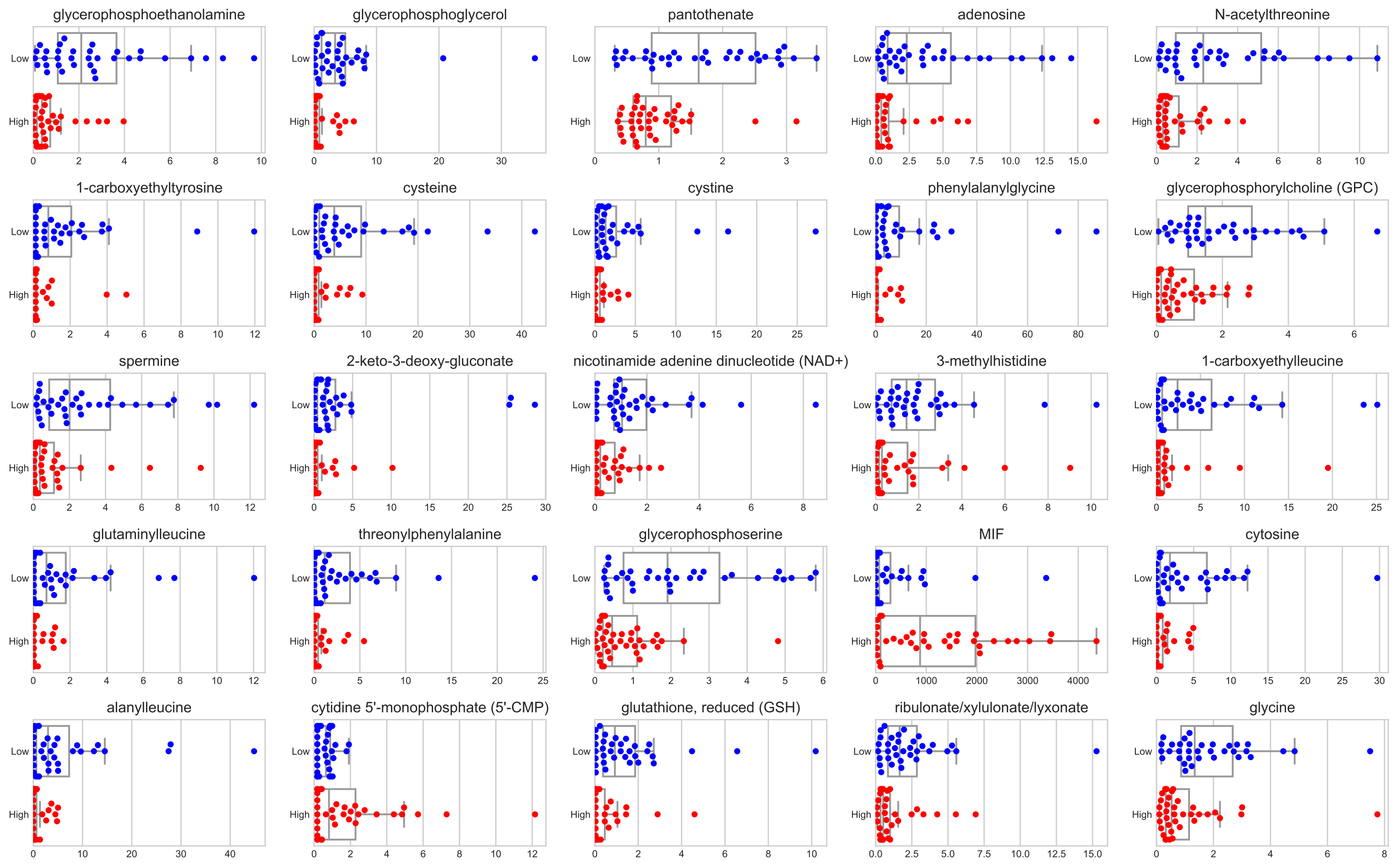

Supplement: S11 Fig — Boxplots display quartile distributions, swarmplots display individual values of top important feature abundances in “typical” (pH ≤ 5.0) and “high” (pH > 5.0) groups. (PDF) [file pcbi.1009876.s013.pdf]

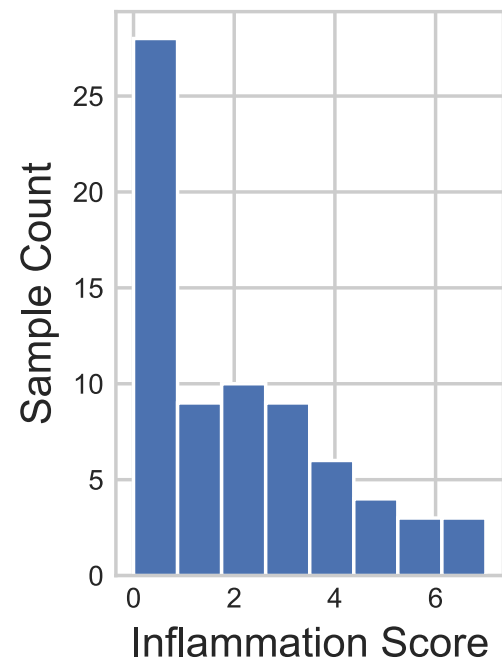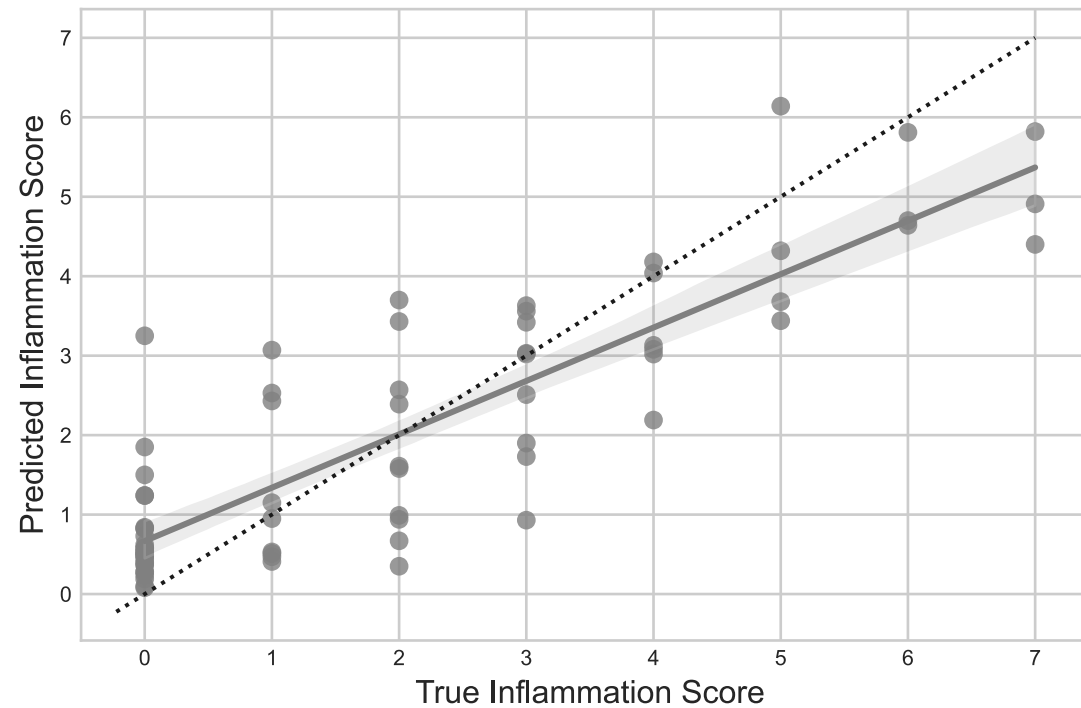

Supplement: S12 Fig — Left, histogram displays number of samples per genital inflammation score. Right, scatterplot displays true vs. predicted inflammation scores for each subject (using 10-fold cross-validation random forest regressors to predict inflammation score across subjects). (PDF) [file pcbi.1009876.s014.pdf]

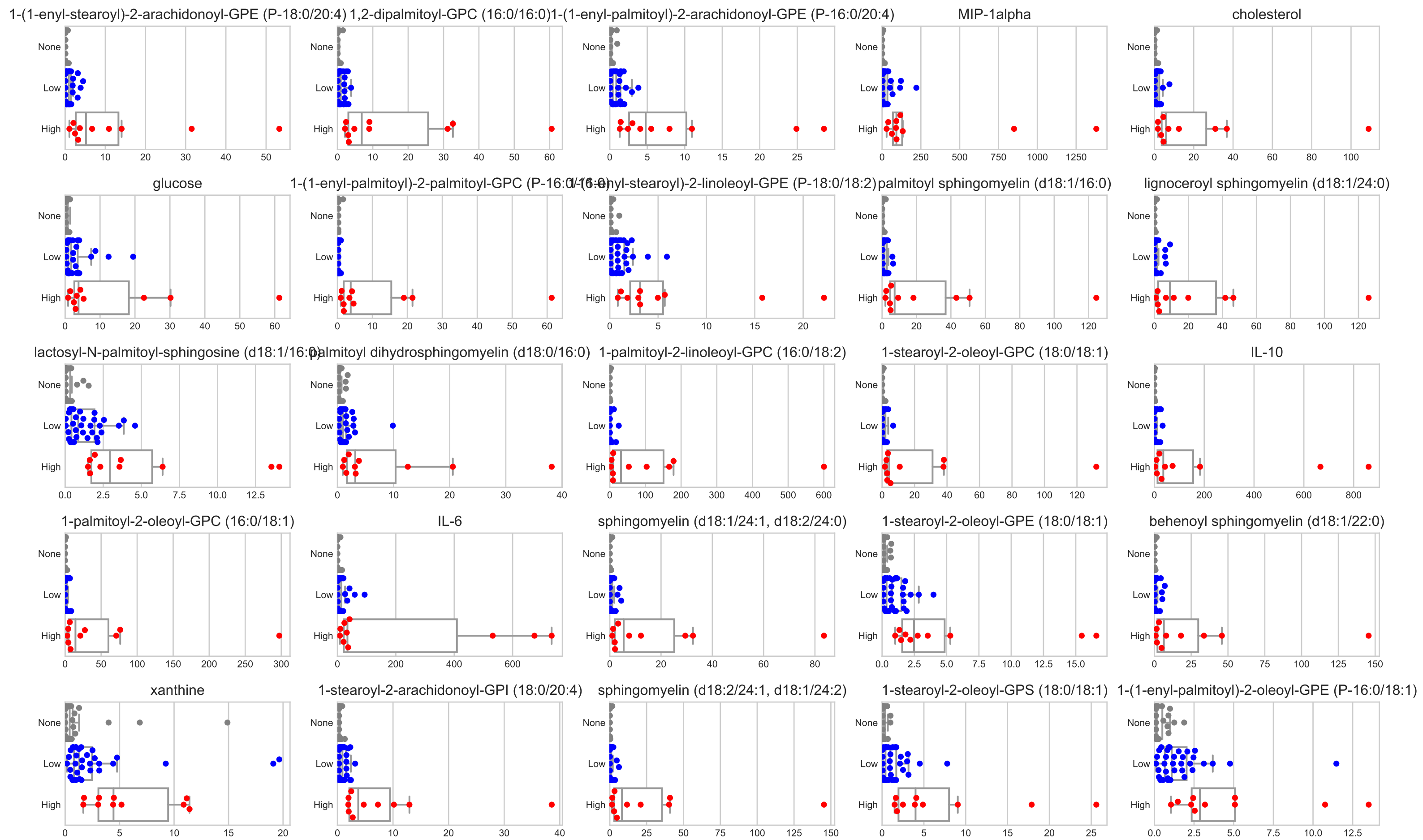

Supplement: S13 Fig — Boxplots display quartile distributions, swarmplots display individual values of top important feature abundances in no (score = 0), low (0 < score < 5), and high inflammation (score ≥ 5) groups. (PDF) [file pcbi.1009876.s015.pdf]
