# Supplementary material for: Multi-omics data integration reveals metabolome as the top predictor of the cervicovaginal microenvironment
Source: PLoS Comput Biol. 2022 Feb 23;18(2):e1009876. doi: 10.1371/journal.pcbi.1009876 (PMC8901057; doi:10.1371/journal.pcbi.1009876)
Supplement: S14 Fig — Receiver operating characteristics (ROC) analysis showing true and false positive rates for each group, using random forest classifiers with 10-fold cross-validation to test predictive accuracy across subjects. Higher area under the curve (AUC) indicates better accuracy. Micro-average is calculated across each sample, and hence impacted by class imbalances. Macro-average gives equal weight to the classification of each sample, eliminating the impact of class imbalances on average AUC. Notably, invasive cervical carcinoma (ICC) cases are predicted moderately well, indicating a characteristic signal associated with ICC but not with intermediate stages of progression. HSIL and LSIL = high- and low-grade squamous intraepithelial lesions, respectively. (PDF) [file pcbi.1009876.s016.pdf]

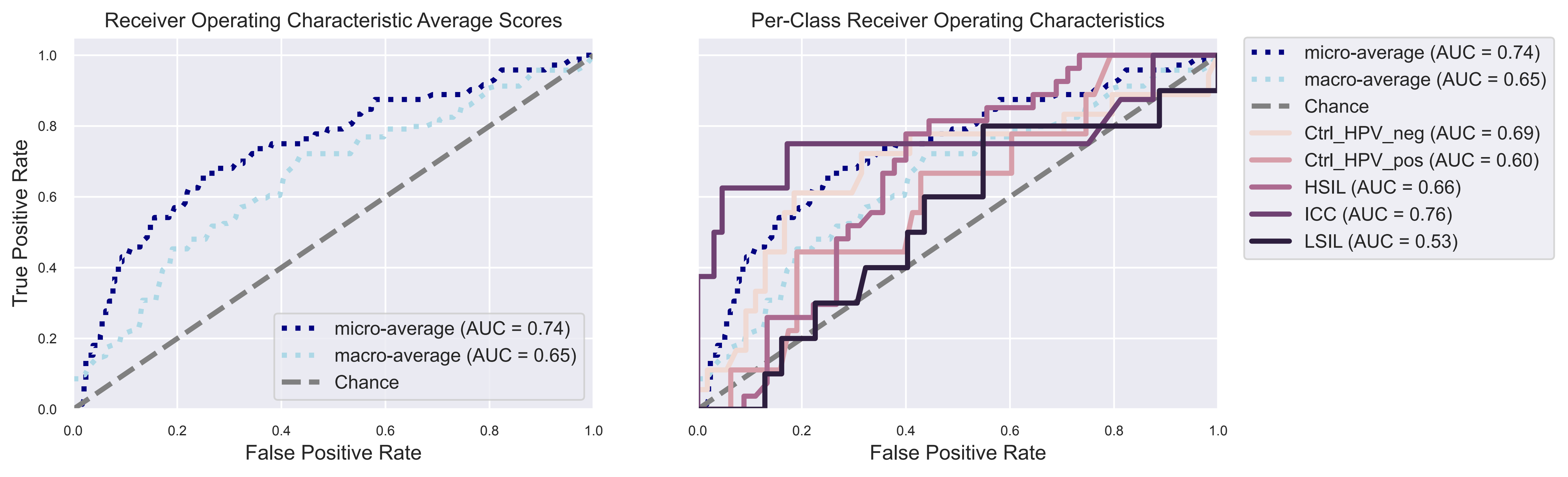
Receiver Operating Characteristic Average Scores
True Positive Rate
False Positive Rate
0.0
0.2
0.4
0.6
0.8
1.0
micro-average (AUC = 0.74)
macro-average (AUC = 0.65)
Chance
Per-Class Receiver Operating Characteristics
False Positive Rate
micro-average (AUC = 0.74)
macro-average (AUC = 0.65)
Chance
Ctrl_HPV_neg (AUC = 0.69)
Ctrl_HPV_pos (AUC = 0.60)
HSIL (AUC = 0.66)
ICC (AUC = 0.76)
LSIL (AUC = 0.53)
