# Supplementary material for: Multi-omics data integration reveals metabolome as the top predictor of the cervicovaginal microenvironment
Source: PLoS Comput Biol. 2022 Feb 23;18(2):e1009876. doi: 10.1371/journal.pcbi.1009876 (PMC8901057; doi:10.1371/journal.pcbi.1009876)
Supplement: S15 Fig — Column names depict selected targets and row names selected omics features. (PDF) [file pcbi.1009876.s017.pdf]

	Lactobacillus dominance	Vaginal pH	Genital inflammation	Disease status
Patient covariates				×
Microbiome		×	×	×
Immuno-proteome	×	×	×[1)]	×
Metabolome	×	×	×	×

1) Excluding seven cytokines and chemokines that were used to define target, namely IL-1α, IL-1β, IL-8, MIP-1β, MIP-3α, RANTES, and TNFα.
