# Supplementary material for: Multi-omics data integration reveals metabolome as the top predictor of the cervicovaginal microenvironment
Source: PLoS Comput Biol. 2022 Feb 23;18(2):e1009876. doi: 10.1371/journal.pcbi.1009876 (PMC8901057; doi:10.1371/journal.pcbi.1009876)
Supplement: S8 Fig — Feature importance of top 20 features used in the final Random Forest regression model for each cancer biomarker prediction displayed in S7 Fig. *Microbial features are displayed in red, with the first 6 characters of the ASV ID followed by the genus/species-level Greengenes taxonomy. (PDF) [file pcbi.1009876.s010.pdf]

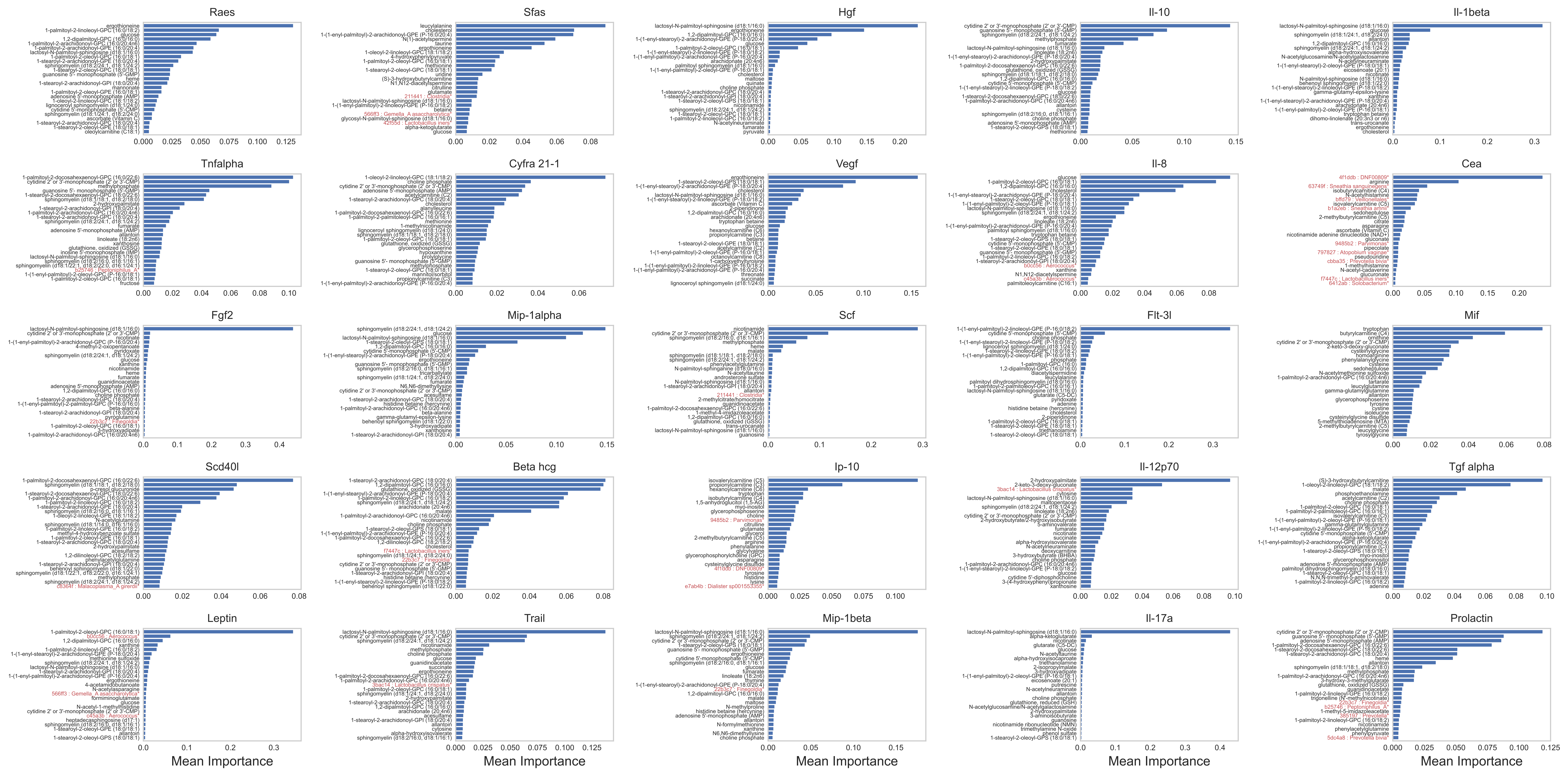
Raes
Sfas
Hgf
Il-10
Il-1beta
Tnfalpha
Cyfra 21-1
Vegf
Il-8
Cea
Fgf2
Mip-1alpha
Scf
Flt-3l
Mif
Scd40l
Beta hcg
Ip-10
Il-12p70
Tgf alpha
Leptin
Trail
Mip-1beta
Il-17a
Prolactin
Mean Importance
Mean Importance
Mean Importance
Mean Importance
Mean Importance
